# Towards a Generalized Bayesian Model of Reconstructive Memory

**DOI:** 10.1007/s42113-024-00222-8

**Published:** 2024-09-27

**Authors:** Zihao Xu, Pernille Hemmer, Qiong Zhang

**Affiliations:** 1 Computer Science Department, Rutgers University–New Brunswick, Piscataway, USA; 2 Psychology Department, Rutgers University–New Brunswick, Piscataway, USA; 3 Center for Cognitive Science, Rutgers University–New Brunswick, Piscataway, USA

**Keywords:** Reconstructive memory, Bayesian modeling, Category effect, Category Adjustment Model, Memory distortion

## Abstract

Prior knowledge has long been known to shape reconstruction from memory. An individual stimulus from a category is often remembered to be closer to the center of that category than its true location. This effect, together with more complex memory effects that involve prior knowledge at multiple levels of abstraction, has been successfully explained by the Category Adjustment Model (CAM; Huttenlocher et al. 2000) and its extensions. However, recent experimental results diverge from CAM’s predictions showing that reconstructive memory for atypical category examples is influenced by the category center less than that of typical category examples. To unify these findings, we propose a generalized Bayesian model of reconstructive memory, called the generalized CAM model (g-CAM). We demonstrate through simulations that g-CAM can account for previously known effects of reconstructive memory, while additionally capturing recent empirical findings involving atypical category examples.

## Introduction

**Fig. 1:**
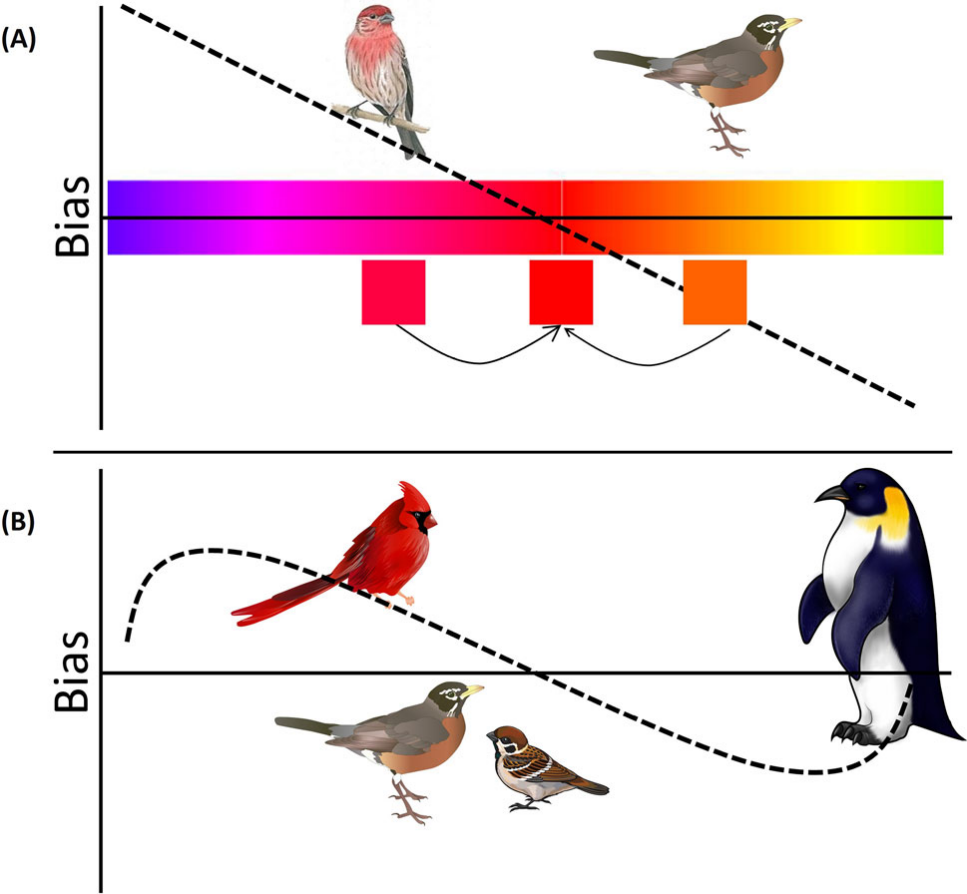
**A** Reconstructive memory effects captured by the Category Adjustment Model (CAM): when you try to recall the color of the robin’s breast you have seen, you might recall it as being a more prototypical red instead of the real orange-red hue. **B** The atypicality effect: when you are walking in the park and see a cardinal, a robin, a sparrow, and a penguin, you are likely to recall having spotted the cardinal (a relatively typical bird) closer to the robin and sparrow than you really did, but you are likely more accurate at recalling the location of the penguin (an atypical bird)

Episodic memory retrieval has been characterized as a process of reconstruction, influenced by one’s prior knowledge (Bartlett, 1932; Brewer & Nakamura, 1984). An individual stimulus from a category is often remembered to be closer to the center of that category than its true location. For example, imagine that you see a robin on your walk in the park. Later, when trying to recall the features of the bird, you recall the color of the breast as being a more prototypical red hue, rather than the orange-red hue it really is (see Fig. 1A for an illustration). In contrast, if you see a House Finch, you might estimate the pinkish color of the breast as being a more red hue. In other words, hue values that are greater than (such as orange-red) or smaller than (such as pinkish-red) the prototypical red are biased towards red. This memory bias towards the category center is thought to be influenced by prior knowledge and expectations and has been demonstrated across different domains of perception and cognition (e.g., Bae et al. (2015); Sarah et al. (2015); Harnad (1987); Liberman et al. (1957); Mitterer and De Ruiter (2008); Bartlett (1932); Persaud and Hemmer (2014); Huttenlocher et al. (2000); see a review, Zhang (2022)). To account for the influence of prior knowledge on reconstruction from memory, Huttenlocher et al. (2000) presented an influential Bayesian model of category effects, the Category Adjustment Model (CAM), which posits that people’s reconstructive memory of noisy stimuli is biased towards a single category mean in order to maximize the average accuracy of their stimulus estimates.

Subsequent extensions to CAM have been proposed to account for more complex effects of reconstructive memory, e.g., when prior knowledge involves multiple levels of abstraction and the different levels have varying degrees of uncertainty (aka the hierarchical memory effects; Hemmer and Steyvers (2009)). However, recent experimental results (Tompary & Thompson-Schill, 2021; Zeng et al., 2021) pose a new challenge for CAM. For instance, Tompary and Thompson-Schill (2021) showed that typical category members are more biased towards their category center than atypical ones, contrary to CAM’s prediction of greater bias for atypical members. These results call for additional updates of the CAM. Given that there are already several extensions to CAM, the question of interest becomes whether a new extension or a new model is needed for each new task, or whether there is a model that can generalize across the observed memory effects. In this paper, we propose a generalized Bayesian model of reconstructive memory (the generalized CAM model or g-CAM) that can capture the recent experimental effect involving atypical examples, while also providing a unified modeling approach to explain effects of reconstructive memory accounted for in previous extensions of CAM.

There are strong reasons for proposing a generalized framework of reconstructive memory. Cognitive scientists are interested in proposing models to account for different experimental effects. However, it would be unrealistic to assume that human cognition needs to switch from one process (under one model) to a completely different process (or model). Newell (1973) argued that with a diverse collection of small experimental tasks, it is challenging to provide the integration that we—as cognitive scientists—seek, and one way to get around that is to develop “one program for many tasks,” i.e., a single system that would be able to perform a set of tasks. Similarly, in constructing Bayesian models of category effects, how convincing these models are depends on not only how well they capture individual experiments, but also on how well they generalize across experiments. In that regard, a model that, with minimal modification, can explain a range of experimental effects is more desirable than a set of models that are each developed for a specific task.

Moving towards this goal, we propose the g-CAM. First, we demonstrate how a special case of this model can capture the category effects on atypical examples. This is a recent empirical finding that diverges from CAM’s predictions. Tompary and Thompson-Schill (2021) demonstrated the atypicality effect in a continuous judgment experiment where participants encoded and retrieved image-location associations. They found that retrieval of locations for typical category members, i.e., stimuli closer to the category center, is more biased to the category center than retrieval for atypical category members, i.e., stimuli far from the category center. For example, imagine again that you are walking in the park. You see a cardinal, and after walking a bit further, you see both a robin and a sparrow. You walk on for quite some time, when you encounter a penguin. Later, you are likely to recall having spotted the cardinal (a relatively typical bird) closer to the robin and sparrow (the most typical birds) than you really did, but you are likely to be more accurate at recalling the location of the penguin (an atypical bird). In fact, the effect appears in the experiments in Huttenlocher’s own work (2000); they found that as the presented stimulus moved further away from the category center, the bias values increased at first but then leveled off or decreased at the boundary of the category distribution. In other words, the bias to the category center is non-linear (see Fig. 1A for an illustration). This is a seemingly opposite effect from the predictions of CAM, namely that the amount of bias towards the category center continuously increases as a target stimulus gets further away from the category center, i.e., the bias towards the category center is linear (Huttenlocher et al., 2000). Huttenlocher and colleagues argued that this result is consistent with their model predictions if one also considers the probability of category membership. Despite all stimuli examples being drawn from the same category, stimuli closer to the category boundary are perceived as belonging less to the category than those that are closer to the category center. However, the probabilistic influence of category membership was not included in the CAM model, and the exact formulation that characterizes the probability of category membership remains to be derived.

The present work generalizes the CAM model to account for these memory effects. Unlike CAM, which assumes that all stimuli must come from a single category, in the generalized case of category effects, we allow multiple categories to exist. This means each stimulus could be drawn from any of these categories, each with its own variance in distribution. Such a generalized form of category effects has been proposed in the speech perception literature and has been successful in explaining a range of categorical effects in phonetic perception (Kronrod et al., 2016; Feldman et al., 2009). We show in the current work that a generalized case of category effects is also useful in unifying results observed in reconstructive memory. However, unlike in the perception literature where categories naturally correspond to sound classes, the novelty of our g-CAM lies in its assumption that *humans are not only actively inferring which category a stimulus belongs to, but also actively inferring which category it does not belong to*. We show that the g-CAM can capture reconstructive memory for atypical examples. Even when there is only one category present in the environment, humans still infer whether a stimulus belongs to that category (i.e., typical examples) or does not belong to that category (atypical examples), with the latter treated in the model as a separate category. Note that this separate category serves as a background category for the purpose of inferring typicality, which is different from the definition of category that is commonly used. Additionally, we show that the g-CAM can capture the hierarchical memory effects with a simpler formulation than the model originally proposed in Hemmer and Steyvers (2009). When the prior knowledge of a category involves multiple levels of abstraction, e.g., an object can be both a “robin” and a “bird,” our generalized framework assumes that humans actively infer whether a stimulus belongs to an object category “robin” or does not belong to “robin.” In the latter case, which is again treated as a separate category, one’s reconstructive memory is primarily influenced by the higher level category “bird.”

In the following sections, we first give a background of the Category Adjustment Model (CAM; Huttenlocher et al. (2000)) and the CAM extension when there are multiple levels of prior knowledge (Hemmer & Steyvers, 2009). We then derive the math for obtaining the general form of the category effects. Next, we run simulations to demonstrate that the g-CAM can capture reconstructive memory of atypical examples in Huttenlocher et al. (2000); Tompary and Thompson-Schill (2021), as well as the hierarchical memory effects in Hemmer and Steyvers (2009). Overall, the present work demonstrates that a single Bayesian framework can account for recent empirical findings on reconstructive memory.

## Background

In this section, we provide an overview of two Bayesian models of reconstructive memory in Huttenlocher et al. (2000) and Hemmer and Steyvers (2009) as well as the key experimental phenomena that they capture.

### The Effect of Categories on Stimulus Judgment

**Fig. 2:**
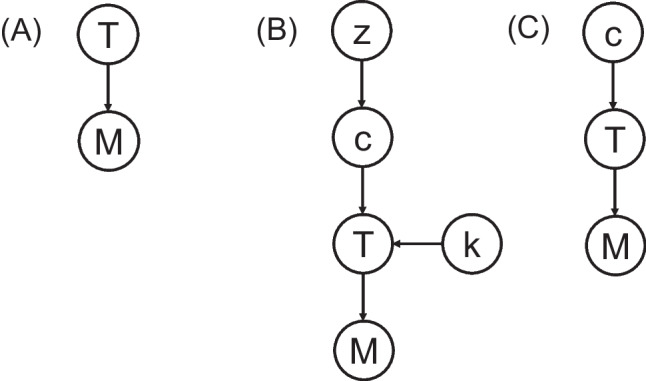
Model structure for **A** the Category Adjustment Model (CAM; Huttenlocher et al. (2000)), **B** the hierarchical memory effect model (Hemmer & Steyvers, 2009), which takes both category priors and object priors into consideration, and **C** our proposed framework g-CAM, where CAM is generalized to multiple categories with any given variance. T: true stimulus value, M: noisy memory, c: category, z: weight on category membership,*k*: prior sample size. Arrows indicate dependencies between random variables. For simplicity, memory noise (variance) is omitted in all three model illustrations

The effect of prior knowledge on judgments for categories—where people’s responses are biased towards the category center when judging stimuli from a given category—is very robust (e.g., Etcoff and Magee (1992); Harnad (1987); Liberman et al. (1957); Mitterer and De Ruiter (2008); Bartlett (1932); Persaud and Hemmer (2014); Huttenlocher et al. (2000); Zhang (2022); Allred et al. (2016); Duffy et al. (2010); Xu and Griffiths (2010); Brady et al. (2018)). Huttenlocher et al. (2000) proposed a Bayesian model of stimulus judgment (aka the Category Adjustment Model or CAM), in which people use their prior knowledge of the stimulus category to adjust the memory of inexactly represented stimuli. For example, Huttenlocher tasked participants with recalling the width of a previously presented image of a fish. CAM provides a rational account of the resulting bias by considering the underlying computational problem of the task and finding the optimal solution to that problem. Specifically, the CAM model considers stimulus reconstruction to be an estimation problem. CAM assumes that the goal of reconstruction is to maximize accuracy, where noisy memory *M* follows a normal distribution with the true stimulus value *T* as mean and $\sigma _T$ as variance, and the category prior follows a normal distribution with mean $\mu _c$ and variance $\sigma _c$ (see Fig. 2A):

1 $$\begin{aligned} T \sim N(\mu _c, \sigma _c^2) \end{aligned}$$

2 $$\begin{aligned} M|T \sim N(T, \sigma _T^2) \end{aligned}$$

Then, the optimal reconstruction of *T* given noisy memory *M* is produced as a weighted sum of the noisy memory trace and the mean of the prior (see details in Huttenlocher et al. (2000)):

3 $$\begin{aligned} \hat{T}=w\mu _c + (1-w)M, \end{aligned}$$

where *w* represents the uncertainty of memory. *w* can be derived by minimizing the mean square difference between the estimation and the true stimulus:

4 $$\begin{aligned} w= \frac{\sigma _{T}^{2}}{\sigma _{T}^{2}+\sigma _{c}^{2}}. \end{aligned}$$

To verify their model, Huttenlocher and colleagues tested participants on stimulus reconstruction with different prior (category) distributions. They found that regardless of the prior, estimation is biased towards the category center, consistent with their model predictions. However, when the prior has a low probabilistic density at the category boundary (e.g., a normal distribution), estimation bias grows less linearly, levels off, or even decreases when a stimulus goes from the category mean to the extreme. The authors added that this resulting non-linear category influence could be reconciled with the model if one takes into account the probability of category membership. In other words, although all stimuli in the experiment were drawn from a single category, stimuli closer to the category boundary could be perceived as belonging less to that category. However, the exact formulation that characterizes the inference of category membership remains to be derived, which we will address in the current work.

### Memory Reconstruction with Multiple Levels of Abstraction: the Hierarchical Memory Effects

The influence of prior knowledge can also come from multiple levels of abstraction. For example, an object can be both an apple (basic-level category) and a fruit (superordinate-level category). Next, we turn to Hemmer and Steyvers (2009) extension of Huttenlocher and colleagues’ (2000) work to account for such hierarchical influences of prior knowledge. Hemmer and Steyvers (2009) asked participants to recall the size of common objects (e.g., fruits, and vegetables). They found that for unfamiliar objects, memory reconstruction is affected mainly by the category prior. Conversely, for familiar objects, reconstruction relies more on the object prior.

To capture the biases from both the category level and the object level (see Fig. 2C), Hemmer and Steyvers (2009) modeled the prior $\mu$, $\sigma$ as a weighted sum of the object prior $\mu _o$, $\sigma _o$ and category prior $\mu _c$, $\sigma _c$:

5 $$\begin{aligned} \mu = z\mu _o + (1-z)\mu _c \end{aligned}$$

6 $$\begin{aligned} \sigma = z\sigma _o + (1-z)\sigma _c \end{aligned}$$

Here, *z* weights the contribution of the object prior versus the category prior and represents the familiarity of an object to a participant. Similar to Huttenlocher et al. (2000), the optimal memory reconstruction is a weighted sum of the noisy memory and the prior mean:

7 $$\begin{aligned} \hat{T} = w'\mu + (1-w')M, \end{aligned}$$

where

8 $$\begin{aligned} w' = \frac{k}{1 + k}. \end{aligned}$$

Here, *k* refers to a constant that is proportional to the prior sample size (Hemmer & Steyvers, 2009). As the prior is estimated with more samples, the reconstruction will be more biased towards the prior. Note that Eq. 7 takes the same form as Eqs. 3 and 18. Therefore, the proposed model can capture the effect of object mean and category mean on memory reconstruction, weighted by the familiarity *z* of the object. The important difference from Huttenlocher et al. (2000) is that memory noise is modeled as an unobserved variable and needs to be coupled with the prior variance of the study event (see details in Hemmer and Steyvers (2009); Fig. 2C). This allows the model to capture a full range of experimental patterns but also significantly complicates the model. In the current work, we will show that our proposed framework g-CAM can capture hierarchical memory effects without imposing additional assumptions such as treating memory noise as an unobserved variable.

## Proposed Framework: g-CAM–A Generalized Bayesian Model of Reconstructive Memory

Humans should be able to use a similar approach to make inference across different tasks and solve the computational problems at hand. Therefore, we aim to unify the original CAM by Huttenlocher et al. (2000) and the hierarchical extension of CAM by Hemmer and Steyvers (2009) into one framework, while additionally showing that this framework captures the reconstructive memory of atypical examples. Essentially, different phenomena can be framed as the same optimal reconstruction process: given noisy content *M*, the goal is to reproduce the target *T* with prior knowledge about multiple prior means $\mu _c$ and variances $\sigma _c$. We assume that people first infer the category of the stimulus and then estimate the target based on the chosen category prior. Formally, if the inferred category *c* has mean $\mu _c$ and variance $\sigma _c$, then the target *T* is generated with $\mu _c$ and $\sigma _c$. The noisy content *M* is generated from *T* with variance $\sigma _T$. The model can be written as (see Fig. 2C)

9 $$\begin{aligned} T|c \sim N(\mu _c, \sigma _c^2) \end{aligned}$$

10 $$\begin{aligned} M|T \sim N(T, \sigma _T^2) \end{aligned}$$

Note that Eqs. 9 and 10 are equivalent to Eqs. 1 and 2 in CAM but do not assume that there is only one possible category *c* as in CAM. The optimal stimulus reconstruction $\hat{T}$ is the mean estimate of *T* given the noisy representation *M*, i.e., *E*[*T*|*M*], which can be written as

11 $$\begin{aligned} \hat{T}= E[T | M]=\sum _{c} E[T | c, M] p (c | M) \end{aligned}$$

An important property of g-CAM is that it can be applied to cases when there are any number of categories with any given variances for their distributions.

To obtain *E*[*T*|*M*], we need to derive two terms: $$E[T | c, $$ $$M]$$ and *p*(*c*|*M*).

The second term *p*(*c*|*M*) can be derived using Bayes’ rule:

12 $$\begin{aligned} p(c|M) = \frac{p(M|c)p(c)}{\Sigma _c p(M,c)} \end{aligned}$$

If we assume *p*(*c*) has a uniform distribution, meaning that a priori all categories are equally likely, then

13 $$\begin{aligned} p(c|M) \propto p(M,c) \end{aligned}$$

where *p*(*M*, *c*) follows a normal distribution (see details of this derivation in Appendix A):

14 $$\begin{aligned} p(M,c)&= \frac{1}{\sqrt{2 \pi } \sqrt{\sigma _{T}^{2}+\sigma _{c}^{2}}} \exp \left( -\frac{(\mu _c-M)^{2}}{2 \sigma _{T}^{2}+2\sigma _{c}^{2}}\right) \nonumber \\ &\quad \sim N\left( \mu _{c}, \sigma _{T}^{2}+\sigma _{c}^{2}\right) \end{aligned}$$

Therefore, we can write

15 $$\begin{aligned} p(c | M&) \propto N\left( \mu _{c}, \sigma _{T}^{2}+\sigma _{c}^{2}\right) \end{aligned}$$

Given the property that $\Sigma _c p(c|M) = 1$, we can find the exact value for *p*(*c*|*M*).

For the first term, we show that $E[T|c,M] = \frac{\mu _{c} \sigma _{T}^{2}+\sigma _{c}^{2}M}{\sigma _{T}^{2}+\sigma _{c}^{2}}$, as *p*(*T*|*c*, *M*) is a normal distribution with mean $\frac{\mu _{c} \sigma _{T}^{2}+\sigma _{c}^{2}M}{\sigma _{T}^{2}+\sigma _{c}^{2}}$ (see Appendix B for full details).

Replacing *E*[*T*|*c*, *M*], we can write *E*[*T*|*M*] as

16 $$\begin{aligned} \hat{T}= E[T| M] =&\Sigma _c E[T| c,M] * p(c|M) \end{aligned}$$

17 $$\begin{aligned} =&\Sigma _c \frac{\sigma _{T}^{2}\mu _{c} +\sigma _{c}^{2}M}{\sigma _{T}^{2}+\sigma _{c}^{2}} * p(c|M) \end{aligned}$$

Equation 17 gives an elegant general form of the category effects—the reconstruction of stimulus *T* is first weighted by the inferred category membership *p*(*c*|*M*), out of any number of possible categories, and under that category membership reconstruction then becomes a weighted combination between the noisy memory *M* and the prior mean $\sigma _{c}$ unique to that category.

The original category adjustment model by Huttenlocher et al. (2000) is a special case of this model when there is no inference of category membership, i.e., $p(c|M)=1$. When every stimulus by default comes from a single category, the reconstruction reduces to

18 $$\begin{aligned} \hat{T}=w\mu _c + (1-w)M, \end{aligned}$$

where

19 $$\begin{aligned} w = \frac{\sigma _{T}^{2}}{\sigma _{T}^{2}+\sigma _{c}^{2}}. \end{aligned}$$

Having derived a generalized formulation in this section, next we will demonstrate with simulations that the g-CAM framework can capture reconstructive memory of atypical examples in both (Huttenlocher et al., 2000; Tompary & Thompson-Schill, 2021), as well as the hierarchical memory effects in Hemmer and Steyvers (2009).

## Simulation 1: Reconstructive Memory of Atypical Examples in Huttenlocher et al. (2000)

**Fig. 3:**
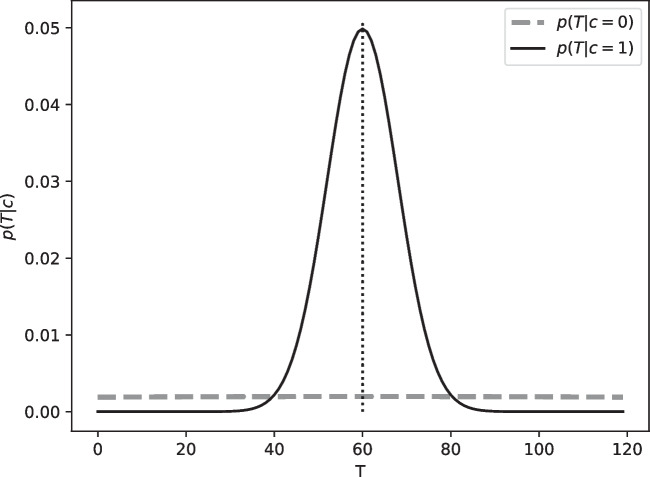
We assume two categories during inference: typical and atypical. Since we have little information for an atypical category, we assume it will exhibit a large variance, essentially approaching a uniform distribution ($p(T|c=0)$ with variance $\sigma _{c=0} = 200$). In contrast, a typical category follows a Gaussian distribution with a much smaller variance ($p(T|c=1)$ with variance $\sigma _{c=1} = 10$)

The key ingredient in our model, for it to capture the experimental results in Huttenlocher et al. (2000), is the assumption that even when there is only one category present (i.e., all examples are drawn from one distribution of stimuli), participants still need to infer which category a stimulus belongs to, i.e., whether a stimulus is an atypical example or typical example of the category. We apply g-CAM by setting one “category” as atypical examples (denoted as $c = 0$) and a second “category” as typical examples (denoted as $c = 1$). Specifically, we have $\sigma _{c=0} \gg \sigma _{c=1}$. The intuition is that when we have little information about atypical items, this greatly increases the variance of the prior distribution. When the variance is large enough, it resembles a uniform distribution (see Fig. 3). Notice that multiple category centers could exist, indicating that *c* has multiple values (e.g., 1, 2, 3, 4...), but only one category, $c = 0$ (the category to which the atypical items belong), has a variance significantly larger than the other categories. This treatment of category in the model, though different from the traditional definition of “category,” is essential for capturing the concept of typicality. Without comparing how much a stimulus comes from a typical category against how much it comes from an atypical category (as well as clearly defining what it means to be considered atypical examples), one cannot determine which examples are more typical than others.

### Results and Discussion

**Fig. 4:**
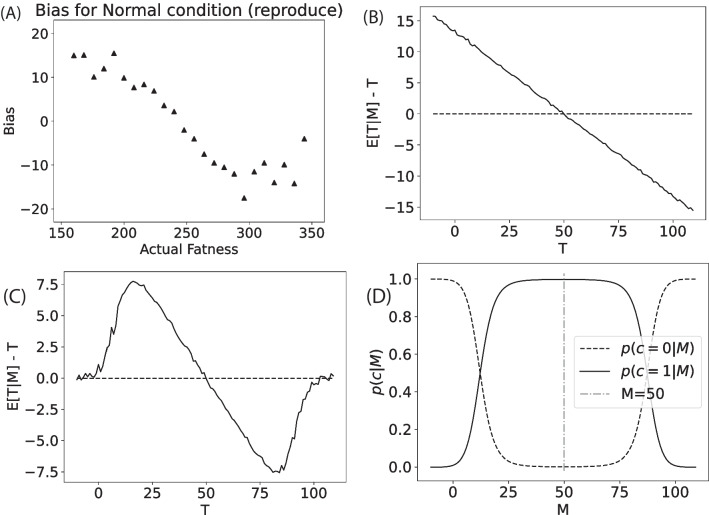
Reconstruction error as a function of stimulus size in Huttenlocher et al. (2000) and model simulations. **A** Reconstruction error reproduced from Fig. 6B in Huttenlocher et al. (2000). **B** Reconstruction error of CAM ($E[T|M] - T$), simulated by reducing g-CAM to a scenario with no inference of category membership. **C** Reconstruction error of g-CAM ($E[T|M] - T$), simulated by having two categories during inference: typical category and atypical category, with$\mu _{c=0}=150$, $\sigma _{c=0} = 200$ and $\mu _{c=1}=50$, $\sigma _{c=1} = 10$. **D** Category membership *p*(*c*|*M*) of g-CAM as a function of the noisy memory *M*, with $\mu _{c=0}=150$, $\sigma _{c=0} = 200$ and $\mu _{c=1}=50$, $\sigma _{c=1} = 10$

Figure 4C shows that simulations from our model align well with the experimental results of Huttenlocher et al. (2000) shown in Fig. 4A. Specifically, g-CAM can capture the observation that biases (i.e., reconstruction errors: $E[T|M] - T$) no longer increase linearly (as CAM would predict in Fig. 4B) at extreme values of stimuli, but rather they flatten off and start to decrease. Figure 4B is when g-CAM is reduced to CAM, with no inference of category membership (i.e., $p(c|M)=1$). To understand this model behavior better, we have shown in Eq. 16 that under multiple categories, the reconstructed estimate can be written as

$$\begin{aligned} E[T| M] = \Sigma _c \frac{\sigma _{T}^{2}\mu _{c} +\sigma _{c}^{2}M}{\sigma _{T}^{2}+\sigma _{c}^{2}} * p(c|M) \end{aligned}$$

In Fig. 4D, we plot *p*(*c*|*M*), showing that at extreme values (when a stimulus *T* is far away from the category center $\mu _{c=1}$), stimuli are categorized as atypical examples, with *E*[*T*|*M*] dominated by $\frac{\sigma _{T}^{2}\mu _{c=0} +\sigma _{c=0}^{2}M}{\sigma _{T}^{2}+\sigma _{c=0}^{2}}$ which is approximately *T* under very large $\sigma _{c=0}$. When the stimulus *T* is closer to the category center $\mu _{c=1}$, stimuli are categorized as typical examples, with *E*[*T*|*M*] dominated by $\frac{\sigma _{T}^{2}\mu _{c=1} +\sigma _{c=1}^{2}M}{\sigma _{T}^{2}+\sigma _{c=1}^{2}}$, which is the same as the Huttenlocher et al. (2000) CAM model.

To summarize, g-CAM more accurately matches the biases at extreme values of stimuli than CAM, by inferring whether these stimuli are typical or atypical examples of the category. The g-CAM has the flexibility to capture the non-linear effect at the boundary of a category. This flexibility comes from the cognitively motivated assumption that people must first infer the category membership of a stimulus—regardless of whether there is one or more categories. In this way, the g-CAM provides a parsimonious explanation that can account for more complex behaviors within a single framework.

**Fig. 5:**
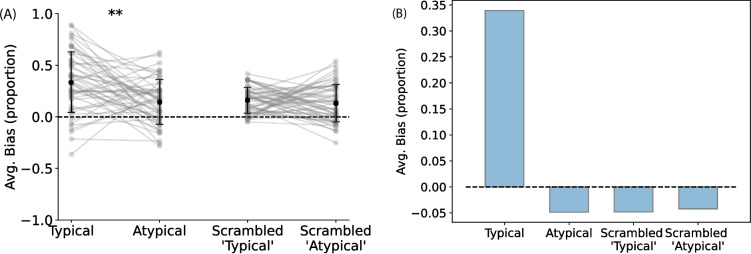
Average bias (proportion) by category typicality and group in Tompary and Thompson-Schill (2021) and in model simulations. **A** Average bias (proportion) by category typicality and group, reproduced from Fig. 2D in Tompary and Thompson-Schill (2021), where $> 0$ indicates retrieval closer to the category center than originally encoded. The control group contains “scrambled typical” and “scrambled atypical” conditions where images were randomly assigned to the locations of typical and atypical category members in the experimental group. **B** Average bias (proportion) by category typicality and group, simulated by g-CAM with two categories: typical category and atypical category, with $\mu _{c=1}=[0,0]$, $\sigma _{c=1} = [1, 1]$ and $\mu _{c=0}=[5,5]$, $\sigma _{c=0} = [200,200]$. For typical images, we assign $p(c=0|V) = 0.05$, $p(c=1|V) = 0.95$, while for atypical images, we assign $p(c=0|V) = 0.95$, $p(c=1|V) = 0.05$. “Scrambled typical” and “scrambled atypical” contain images that no longer cluster by category and are simulated with the same parameters except for having large variances for both typical and atypical categories $\sigma _{c=0} = \sigma _{c=1} = [200,200]$. Additional details for the simulation reproduction can be found in Appendix D

## Simulation 2: Reconstructive Memory of Atypical Examples in Tompary and Thompson-Schill (2021)

Having illustrated how g-CAM can account for reconstructive memory of atypical examples observed in Huttenlocher et al. (2000), next, we turn to a different scenario of atypical examples as set up in Tompary and Thompson-Schill (2021). The study by Tompary and Thompson-Schill (2021) examines how category typicality affects distortions in episodic memory. While the reconstructive memory is along the same dimension as stimulus typicality in Huttenlocher et al. (2000) (i.e., size of the stimulus is both what determines category membership and what is eventually reconstructed), the study by Tompary and Thompson-Schill (2021) dissociated the two dimensions in an experiment where participants encoded and retrieved image-location associations. While visual information in the images determined category membership (e.g., an image of a cardinal is a typical example of a bird category, whereas an image of an ostrich is an atypical example of a bird category), 2D locations on a grid were reconstructed by the participants for a given image. This leads to a novel inquiry into how memory for the spatial location of a stimulus is biased by integrating visual information about its category typicality. In the experiment, images belonging to the same category were generally positioned close to one another. This enabled participants to encode location information with the help of category membership. Their findings revealed that randomly placed images, if considered typical of their category, were more likely to be biased towards the central location of their category than those deemed atypical.

Generalizing the same g-CAM framework to two dimensions to represent a 2D location in a grid, we propose that individuals initially assess the category of a stimulus *p*(*c*|*V*) based on the visual appearance of the objects *V*, with *P*(*o*|*M*) in Eq. 21 replaced by *P*(*o*|*V*), and subsequently estimate the target memory based on the selected category’s prior:

20 $$\begin{aligned} E[T | M]=\sum _{o} E[T | c, M] p (c | V) \end{aligned}$$

Note that now *T* is a 2-dimension variable. To model the judgment of category membership based on visual cues (e.g., one can identify an ostrich as an atypical example from the image and one’s own semantic knowledge), we assign typical items a higher probability of belonging to the typical category based on visual cues, expressed as $p(c=1|V)$, while atypical items are assigned a higher probability of being associated with a background atypical category, indicated by $p(c=0|V)$. This setup is similar to the one in Simulation 1 but allows us to extend our model (1) from 1D stimulus to 2D stimulus, and (2) to a scenario where *p*(*c*|*V*) is not inferred from the stimulus dimension itself *M* but from visual cues *V* and one’s semantic knowledge. Full implementation details of the simulation can be found in Appendix D.

### Results and Discussion

Figure 5B shows that simulations from our model align well with the experimental results of Tompary and Thompson-Schill (2021) that we reproduced in Fig. 5A. Specifically, g-CAM is able to capture the observation that randomly placed items, if considered “typical” of their category, were more biased towards the central location of their category than those identified as “atypical” (Fig. 5A). There is no difference between “scrambled typical” and ‘scrambled atypical” in the control condition, created by having the same locations as those in the experimental group but the images did not cluster by category (randomly assigned). This result is intuitive from a rational perspective. In the experimental condition, images cluster by category. When a stimulus is considered a typical example of a category, biasing towards the category center is optimal, as the category information can help reduce the uncertainty in the reconstructed memory. When a stimulus is considered an atypical example of a category, biasing toward the category center is no longer desired. In the control condition, since images no longer cluster by category, the category knowledge of images is not useful for reconstructing image locations; therefore, there is no difference in the bias towards category center between “scrambled typical” and ‘scrambled atypical” conditions. There is a discrepancy between our simulation and the experimental results: the average biases in the atypical and scrambled conditions in our simulations were slightly negative, in contrast to the positive biases observed by Tompary and Thompson-Schill (2021). This discrepancy might stem from the original experiment’s use of a grid layout on the screen for encoding and retrieving locations. Such a setup likely introduced an inherent bias, causing items to be remembered as closer to the center of the grid. Despite this difference, our findings still support the conclusion that category typicality affects recall biases.

To summarize, g-CAM can capture the effect of category membership on memory distortions of image locations in Tompary and Thompson-Schill (2021), by inferring whether these stimuli are typical or atypical examples of the category. This further illustrates the flexibility and parsimony of g-CAM. This single framework captures the influence of a single category on a stimulus (as in CAM), the reconstructive memory of atypical examples when they lie at extreme values (Huttenlocher et al., 2000), as well as capturing atypical examples for two-dimensional stimuli in a situation where category inference and stimulus reconstruction is disassociated (Tompary & Thompson-Schill, 2021). Lastly, we demonstrate another flexibility of the model: inferring category membership and familiarity when prior knowledge involves multiple levels of abstraction.

## Simulation 3: The Hierarchical Category Effect

Human’s prior knowledge can have multiple levels of abstraction. In Hemmer and Steyvers (2009), participants were asked to recall the size of common objects (e.g., fruits and vegetables). When studying a specific stimulus, e.g., an apple, participants were assumed to have information about features of that object drawn from an object-specific prior. These features were in turn drawn from a more general—or superordinate-level category—of fruits, which was assumed to have a category-specific prior. Hemmer and Steyvers (2009) built a Bayesian model that integrates the effect of the object prior versus the category prior by modeling the prior mean $\mu$ and variance $\sigma$ as a weighted sum of the object prior $\mu _o$, $\sigma _o$ and category prior $\mu _c$, $\sigma _c$, such that $\mu = z\mu _o + (1-z)\mu _c$, $\sigma = z\sigma _o + (1-z)\sigma _c$, where *z* represents familiarity with the object. Unlike a fixed memory noise in Huttenlocher et al. (2000), the memory noise $\sigma _m$ is treated as a latent variable to be inferred. Figure 2B illustrates the probabilistic graphic model of this framework.

The Hemmer and Steyvers (2009) model could be reformulated into a simpler model, following our general scheme. The simplified model follows the assumption that when people are familiar with the object, they will do reconstruction mainly based on the object level prior. Otherwise, they will depend on the category level prior more.

Using the same g-CAM framework, we assume people first infer the category of the stimulus, *o*, and then estimate the target memory based on the chosen category prior:

21 $$\begin{aligned} E[T | M]=\sum _{o} E[T | o, M] p (o | M) \end{aligned}$$

Here, we refer to the category as *o* instead of *c*, as it represents whether a stimulus is considered as part of the object membership (i.e., $o=1$) versus not part of the object membership (i.e., $o=0$).

There is rich hierarchical information in the stimuli used in Hemmer and Steyvers (2009). When a stimulus is identified as part of the object membership, e.g., an apple, the recall will be biased based on the prior knowledge about apples, $T|o=1 \sim N(\mu _o, \sigma _o^2)$:

22 $$\begin{aligned} E[T \mid o=1, M]=\frac{M\sigma _{o}^{2}+\mu _{o} \cdot \sigma _{T}^{2}}{\sigma _{o}^{2}+\sigma _{T}^{2}} \end{aligned}$$

When a stimulus is identified as not being from the object membership, identification will resort to the prior information from the higher-level category, e.g., fruits, $T|o=0 \sim N(\mu _c, \sigma _c^2)$:

23 $$\begin{aligned} E[T \mid o=0, M]=\frac{M\sigma _{c}^{2}+\mu _{c} \cdot \sigma _{T}^{2}}{\sigma _{c}^{2}+\sigma _{T}^{2}} \end{aligned}$$

In other words, instead of combining the object prior and the category prior at the level of the prior mean and prior variances, as was formulated in Hemmer and Steyvers (2009), we adopt the same g-CAM model as in Simulation 1 and 2. Here, people first infer the category of the stimulus, then reconstruct the target based on the chosen category prior. In this case, the inferred category is either part of an object membership or not. This setup resembles Simulation 1 where the goal is to infer whether an example is part of the category or not. Different than that in Simulation 1, if an example does not possess the inferred object membership, it will be assumed to have the prior information of a category instead of the prior information of atypical examples.

The stimuli used in the experiment conducted by Hemmer and Steyvers (2009) were colorful images with rich visual details. Even though the measure to reconstruct is the size of the stimulus *T*, it is more likely that participants infer the stimulus category based on rich visual information, e.g., color and shape, rather than the size information of the stimulus alone. To capture this intuition, we replace *P*(*o*|*M*) in Eq. 21 with *P*(*o*|*V*), where *V* represents the visual details of the stimulus:

24 $$\begin{aligned} E[T | M, V]=\sum _{o} E[T | o, M] p (o | V) \end{aligned}$$

*p*(*o*|*V*) naturally represents one’s “familiarity” with the object and can be replaced with $\alpha$ using the same annotation as Hemmer and Steyvers’ model:

25 $$\begin{aligned} E[T | M, V]=\alpha E[T|o=1,M] + (1 - \alpha ) E[T|o=0,M] \end{aligned}$$

When there is high familiarity with the object (large $\alpha$), i.e., the probability of identifying the object given the visual image is high, participants mainly rely on the reconstruction based on one specific object. Otherwise, participants rely on the prior knowledge of the overall category.

**Fig. 6:**
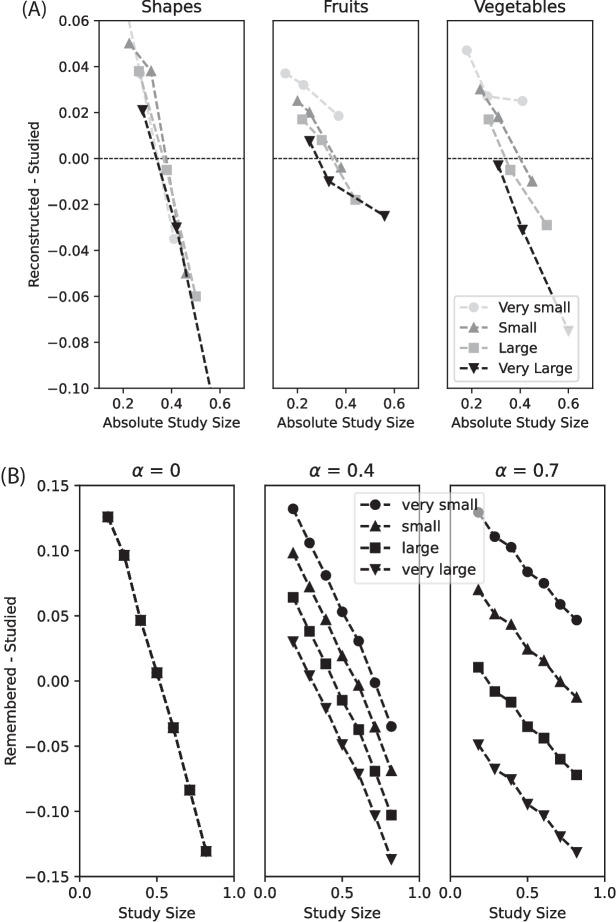
Reconstruction error as a function of category and object in Hemmer and Steyvers (2009) and in model simulations. **A** Empirical reconstruction error reproduced from Fig. 5 of Hemmer and Steyvers (2009). Study objects were divided into four classes relative to the mean of the object. Negative slopes show correction toward the category center and are indicative of category-level knowledge effects. Intercept differences by relative study size are indicative of object-level knowledge effects. **B** Reconstruction error $\alpha E[T|M] + (1 - \alpha ) E[T'|M]) - T$ in simulations as a function of the study size $T$, with varied familiarity $\alpha$

### Results and Discussion

The g-CAM model is able to capture the main experimental effects of Hemmer and Steyvers (2009) (illustrated in Fig. 6A). In their experiment, three categories (“shapes,” “fruits,” “vegetables”) of study objects were divided into four classes based on the study sizes relative to the typical size of that object. The difference in intercepts by study size supports the prediction of object prior effects, observed in stimuli of “fruits” and stimuli of “vegetables” but not in stimuli of “shapes.” The non-zero slope supports the prediction of category prior effects, observed in all three types of stimuli. The experimental patterns show that memory is biased towards both the object mean and the category mean when there is familiarity with objects (“fruits,” “vegetables”) and biased towards the category mean only when there is no familiarity with objects (“shapes”).

The g-CAM simulations shown in Fig. 6B capture this effect of familiarity on category bias. A key effect to observe is that as familiarity $\alpha$ increases, the difference in intercepts by study size increases, i.e., increased influence from the object prior, while the difference in the slopes decreases, i.e., decreased influence from the category prior.

The advantages of our model, compared with Hemmer and Steyvers’ model in capturing these experimental results, lie in (1) its simplicity, without the need to infer the memory noise, (2) its consistency with how atypical item effects are captured using the same modeling framework, and (3) the intuitive interpretation of “familiarity,” as the probability of identifying an object given the visual information.

## Conclusions

Prior knowledge influences the reconstruction of stimulus from memory. Models characterizing the effect have also proliferated over the decades, including the influential Category Adjustment Model (CAM; Huttenlocher et al., 2000) and extensions of CAM to account for more complex effects of category bias. Recent experimental results diverged from CAM’s predictions by demonstrating a smaller memory bias towards the category center for atypical examples than that for typical examples. Here, we unified these findings by introducing a generalized Bayesian model of reconstructive memory, called the generalized CAM model (g-CAM). In the generalized case of reconstructive memory, we assume that reconstruction from memory could be influenced by any number of categories, with any given variances for their distributions, and that humans actively infer which category a stimulus belongs to even when there is only one category in the environment. We demonstrated through simulations that the g-CAM model can account for previously known effects of reconstructive memory in Huttenlocher et al. (2000) and Hemmer and Steyvers (2009) that have been captured by CAM and its extensions, as well as empirical findings involving atypical examples that diverged from predictions of CAM. Furthermore, the model is nested such that when there is only one category, it is the same as the original CAM model. This generalized model provides a parsimony that integrates known memory effects into a single framework.

## Data Availability

Availability of data and material is not applicable in this study.

## A. Calculation of *p*(*c*|*M*)

A.1 $$\begin{aligned} \begin{aligned} {\begin{matrix} p(M, c) & \!=\!\int _{T} p(c, M, T) dT \\ & \!=\! \int _{T} P(T | c) p(M | T) dT \\ & \!=\! \int _{-\infty }^{+\infty } \frac{1}{2 \pi \sigma _{T} \sigma _{c}} \!\exp \! \left( -\frac{(T-\mu _c)^{2}}{2 \sigma _{c}^{2}}-\frac{(M-T)^{2}}{2 \sigma _{T}^{2}}\right) dT \end{matrix}} \end{aligned} \end{aligned}$$

A.2 $$\begin{aligned} \begin{aligned} {\begin{matrix} p(M, c) & \!=\!\int _{T} p(c, M, T) dT \\ & \!=\! \int _{T} P(T | c) p(M | T) dT \\ & \!=\! \int _{-\infty }^{+\infty } \frac{1}{2 \pi \sigma _{T} \sigma _{c}} \!\exp \! \left( -\frac{(T-\mu _c)^{2}}{2 \sigma _{c}^{2}}-\frac{(M-T)^{2}}{2 \sigma _{T}^{2}}\right) dT \end{matrix}} \end{aligned} \end{aligned}$$

Given the fact that the following formula holds for $\forall a \in \mathbb {R^+}, b \in \mathbb {R}, C \in \mathbb {R}$ (Adindu-Dick, 2022):

A.3 $$\begin{aligned} \int _{-\infty }^{+\infty }e^{-ax^2 - bx - m} dx = \frac{\sqrt{\pi } e^{\frac{b^2-4am}{4a}}}{\sqrt{a}} \quad (a>0) \end{aligned}$$

From Eq. A.2, we can derive that

A.4 $$\begin{aligned} a = \frac{1}{2\sigma _c^2} + \frac{1}{2\sigma _T^2}, b= -\frac{\mu _c}{\sigma _c^2} - \frac{M}{\sigma _T^2}, m = \frac{\mu _c^2}{2\sigma _c^2} + \frac{M^2}{2\sigma _T^2} \end{aligned}$$

Combining (A.3) and (A.4), we can derive *p*(*M*, *c*) and *p*(*c*|*M*).

A.5 $$\begin{aligned} p(M,c)&\!=\!\int _{-\infty }^{+\infty } \frac{1}{2 \pi \sigma _{T} \sigma _{c}} \exp \left( -\frac{(T-\mu _c)^{2}}{2 \sigma _{c}^{2}}-\frac{(M-T)^{2}}{2 \sigma _{T}^{2}}\right) d T \nonumber \\ =&\frac{1}{2 \pi \sigma _{T} \sigma _{c}} \frac{\sqrt{\pi }}{\sqrt{\frac{1}{2 \sigma _{T}^{2}}+\frac{1}{2 \sigma _{c}^{2}}}} \exp \left( -\frac{(\mu _c-M)^{2}}{2 \sigma _{T}^{2}+2 \sigma _{c}^{2}}\right) \nonumber \\ =&\frac{1}{\sqrt{2 \pi } \sqrt{\sigma _{T}^{2}+\sigma _{c}^{2}}} \exp \left( -\frac{(\mu _c-M)^{2}}{2 \sigma _{T}^{2}+2\sigma _{c}^{2}}\right) \nonumber \\ p(c | M&) \propto N\left( \mu _{c}, \sigma _{T}^{2}+\sigma _{c}^{2}\right) \end{aligned}$$

## Appendix B. Calculation of *E*[*T*|*c*, *M*]

To calculate *E*[*T*|*c*, *M*], we will prove that *p*(*T*|*c*, *M*) is a normal distribution, with a mean of $\frac{\left( \mu _{c} \sigma _{T}^{2}+\sigma _{c}^{2}M\right) }{\sigma _{T}^{2}+\sigma _{c}^{2}}$.

Notice that the generative process in Eqs. 1 and 2 is a typical normal-normal model; we can obtain the distribution analytically with the following deduction.

Firstly, we will decompose *p*(*T*|*c*, *M*) using the Bayes rule. Since we assume that *p*(*c*) has a uniform prior distribution and the likelihood *p*(*c*, *M*) does not change with respect to the variable *T*, the posterior distribution is proportional to *p*(*M*|*T*)*p*(*T*|*c*).

A.6 $$\begin{aligned} p(T| M,c)&=\frac{p(M| T) p(T|c)p(c)}{p(M,c)} \propto p(M| T)p(T| c) \end{aligned}$$

Now, we further plug in the parameters $\sigma _c, \mu _c, \sigma _T$ in Eqs. 1 and 2 into the normal distribution formula, and we obtain the expression of the posterior distribution as shown in Eq. A.7:

A.7 $$\begin{aligned} \begin{aligned} p(T| c)&=\frac{1}{\sqrt{2\pi \sigma _{c}^{2}}}\exp \left\{ -\frac{(T-\mu _{c})^{2}}{2 \sigma _{c}^{2}}\right\} \\ p(M| T)&=\frac{1}{\sqrt{2\pi \sigma _{T}^{2}}}\exp \left\{ -\frac{(M-T)^{2}}{2 \sigma _{T}^{2}}\right\} \\ p(T| M,c)&\propto p(M| T) p(T| c) \\&= \frac{1}{\sqrt{\sigma _{c}^{2}\sigma _{T}^{2}}}\exp \left\{ \frac{-(T-\mu _{c})^{2}}{2\sigma _{c}^{2}}+\frac{-(T-M )^{2}}{2 \sigma _{T}^{2}}\right\} \end{aligned} \end{aligned}$$

We can then convert the term inside the exponential function to a quadratic form shown in Eq. A.8:

A.8 $$\begin{aligned}&\!=\!\exp \left\{ -\frac{1}{2}\left( \frac{T^{2}-2 \mu _{c} T+\mu _{c}^{2}}{\sigma _{c}^{2}}+\frac{M^{2}-2 M T+T^{2}}{\sigma _{T}^{2}}\right) \right\} \nonumber \\&\!=\!\exp \left\{ -\frac{1}{2}\frac{\sigma _{T}^{2} T^{2}-2 \mu _{c} \sigma _{T}^{2} T+\mu _{c}^{2} \sigma _{T}^{2}+\sigma _{c}^{2} M^{2}-2 \sigma _{c}^{2} M T+\sigma _{c}^{2} T^{2}}{\sigma _{c}^{2}+\sigma _{T}^{2}} \right\} \nonumber \\&\!\propto \! \exp \left\{ \frac{\left( \sigma _{T}^{2}+\sigma _{c}^{2}\right) T^{2}-2\left( \mu _{c} \sigma _{T}^{2}+\sigma _{c}^{2} M\right) T}{\sigma _{c}^{2}+\sigma _{T}^{2}}\right\} \nonumber \\&\!\propto \! \exp \left\{ \frac{\left( T-\frac{\left( \mu _{c} \sigma _{T}^{2}+\sigma _{c}^{2} M\right) }{\sigma _{T}^{2}+\sigma _{c}^{2}}\right) ^{2}}{\frac{\sigma _{c}^{2} \sigma _{T}^{2}}{\sigma _{ M}^{2}+\sigma _{c}^{2}}}\right\} \end{aligned}$$

This concludes the proof that the distribution of *p*(*T*|*c*, *M*) is a normal distribution, with mean $\frac{\mu _{c} \sigma _{T}^{2}+\sigma _{c}^{2}M}{\sigma _{T}^{2}+\sigma _{c}^{2}}$.

### Appendix C. Sampling Strategies of All Simulations

Using Eq. 16, we need both the noisy memory *M* and the category center $\mu _c$ to determine the target *T*. However, while we do not have direct access to human memory, we do know the target *T*. To simulate the reconstruction process, we sample *M* from the normal distribution $N(T, \sigma _T)$, and then use such *M* to estimate the expectation of *T* given noisy memory *M* with Eq. 16.

### Appendix D. Details of Simulation 2 (Fig. 5B)

To calculate bias as described by Tompary and Thompson-Schill (2021), three key elements are required: the cluster center’s location, the encoded (true) location of the object, and the (biased) retrieved location. To determine these elements, we follow these steps:

- Define the cluster center $\mu _{c=1}$: Set the cluster center at $\mu _{c=1}=[0,0]$, with variance $\sigma _{c=1} = [1, 1]$. This is a 2-dimensional Gaussian distribution with independent dimensions (covariance = 0).
- Sample the encoded location *T*: Randomly sample points within a radius of 2 from the cluster center to use as the encoded locations.
- Generate the noisy memory *M*: Use the encoded location *T* as a new center to sample additional points. These points, representing noisy memories *M*, are drawn from a 2-dimensional Gaussian distribution with independent dimensions and a variance of $\sigma _{T} = [0.3, 0.3]$.
- Estimate the retrieved location *E*[*T*|*M*]: Using previous settings and additional parameters like the mean and variance of the atypical item center and the likelihood of items being typical/atypical, apply Eq. 17: $E[T|M] = \Sigma _c \frac{\sigma _{T}^{2}\mu _{c} +\sigma _{c}^{2}M}{\sigma _{T}^{2}+\sigma _{c}^{2}} * p(c|M)$ to estimate the expected true location of the object, which will act as the retrieved location.

With the cluster center $\mu _{c=1}$, the encoded (true) location *T*, and the retrieved (biased) location *E*[*T*|*M*] determined, the biases *d* can now be calculated using the formula provided in Tompary and Thompson-Schill (2021):

$$\begin{aligned} d = \frac{\sqrt{\Vert T - \mu _{c=1}\Vert _2} - \sqrt{\Vert E[T|M] - \mu _{c=1}\Vert _2}}{\sqrt{\Vert E[T|M] - T\Vert _2}}. \end{aligned}$$

## Code Availability

All simulation code supporting this manuscript can be found at https://github.com/shsjxzh/GBMCE.
