# Correction: Towards a Generalized Bayesian Model of Reconstructive Memory

**DOI:** 10.1007/s42113-024-00226-4

**Published:** 2024-11-06

**Authors:** Zihao Xu, Pernille Hemmer, Qiong Zhang

**Affiliations:** 1 Computer Science Department, Rutgers University–New Brunswick, Piscataway, USA; 2 Psychology Department, Rutgers University–New Brunswick, Piscataway, USA; 3 Center for Cognitive Science, Rutgers University–New Brunswick, Piscataway, USA

**Correction: Computational Brain & Behavior**

10.1007/s42113-024-00222-8

The original online version of this article was revised to remove a superfluous subtitle and to correct the format of a reference in the abstract.

The original article has been corrected.

